# Serum GRP78 as a Tumor Marker and Its Prognostic Significance in Non-Small Cell Lung Cancers: A Retrospective Study

**DOI:** 10.1155/2015/814670

**Published:** 2015-07-21

**Authors:** Xiao Ma, Wei Guo, Su Yang, Xiaoli Zhu, Jiaqing Xiang, Hecheng Li

**Affiliations:** ^1^Department of Thoracic Surgery, Fudan University Shanghai Cancer Center, Shanghai, China; ^2^Department of Oncology, Shanghai Medical College, Fudan University, Shanghai 200032, China; ^3^Department of Thoracic Surgery, Nanjing Chest Hospital, Nanjing 210029, China; ^4^Department of Pathology, Fudan University Shanghai Cancer Center, Shanghai 200032, China; ^5^Department of Thoracic Surgery, Ruijin Hospital, Shanghai Jiaotong University School of Medicine, Shanghai 200025, China

## Abstract

*Introduction*. Glucose-regulated protein 78 (78 kDa, GRP78), which is also known as immunoglobulin heavy chain binding protein (BIP), is a major chaperone in the endoplasmic reticulum (ER). The expression and clinical significance of GRP78 in the serum of non-small cell lung cancer patients have not yet been clearly described. The aims of the present study were to investigate the expression of GRP78 in the serum of non-small cell lung cancer patients, the relationships with clinicopathological parameters, and the potential implications for survival. *Patients and Methods*. A total of 163 peripheral blood samples from non-small cell lung cancer patients were prospectively collected at the Department of Thoracic Surgery, Fudan University Shanghai Cancer, China. Clinical characteristics data, including age, gender, stage, overall survival (OS) time, and relapse-free survival (RFS) time, were also collected. Serum GRP78 levels were measured using a commercially available ELISA kit. The associations between GRP78 levels and clinicopathological characteristics and survival were examined using Student's *t*-test, Kaplan-Meier, or Cox regression analyses. *Results*. The mean ± standard error (SE) value of GRP78 was 326.5 ± 49.77 pg/mL. This level was significantly lower compared with the level in late-stage non-small cell lung cancer patients (1227 ± 223.6, *p* = 0.0001). There were no significant correlations with the clinicopathological parameters. No significant difference was found between high GRP78 expression and low GRP78 expression with regard to RFS (*p* = 0.1585). However, the OS of patients with higher GRP78 expression was significantly poorer (*p* = 0.0334). *Conclusions*. GRP78 was expressed in non-small cell lung cancer patients and was highly enriched in late-stage lung cancer. GRP78 may have an important role in the carcinogenesis of non-small cell lung cancer and may be a prognostic marker for non-small cell lung cancer.

## 1. Background

Lung cancer remains the leading cause of cancer-related deaths worldwide. Despite improved multidisciplinary treatments, the overall 5-year survival rate is only approximately 15% [1, 2]. Most lung cancer patients have advanced stage disease at diagnosis, which is often associated with metastasis and a poor prognosis [3–5]. More information is needed to predict the prognosis of patients. Molecular diagnostics may offer objective and systematic human cancer classification. However, the molecular marker standards for human cancers that can be used to predict prognosis have not yet been identified [6].

Glucose-regulated protein 78 (78 kDa, GRP78) is well established as an ER chaperone and widely used as a marker for ER stress [7]. Large amounts of GRP78 were found to be secreted by various tumor cell types [8]. Under stressful conditions such as low glucose, low oxygen, and low calcium ion concentrations, GRP78 is highly expressed for the purpose of maintaining ER stability and cell protection [9, 10]. GRP78 is closely related to carcinogenesis, development, and differentiation [11, 12]. As a potential marker to predict the prognosis of patients with lung cancer, the expression of serum GRP78 has not yet been clearly described. The clinical significance of GRP78 in patients with non-small cell lung cancer also has not been fully investigated.

With these notions mentioned above, we thus measured the serum GRP78 levels and investigate its predictive value for the prognosis of patients with lung cancer.

## 2. Material and Methods

### 2.1. Sample Selection

The study protocol was approved by the institutional review board of Fudan University Shanghai Cancer Center, China. Patients with lung cancer who were treated at the cancer center from April 2009 to September 2014 were enrolled in the study. The diagnosis of lung cancer was confirmed using histopathology in the surgical group. Pathological staging was performed according to the TNM classification of the 7th edition of the American Joint Committee for Cancer Staging manual guidelines [13].

### 2.2. Enzyme-Linked Immunosorbent Assay (ELISA)

Peripheral blood (5 mL) was taken from subjects of each group without the addition of anticoagulant and was allowed to clot for 2 h at room temperature. Each sample was then centrifuged for 20 min at 1,000 ×g. The serum was separated and stored in small aliquots at −20°C until further protein analysis.

Serum GRP78 levels were measured using a commercially available ELISA Kit (GRP78/BiP ELISA kit, Enzo Life Sciences, ADI-900-214), per the manufacturer's protocol.

### 2.3. Clinical Variables and Follow-Up

Clinical variables collected included age at diagnosis, gender, tumor differentiation, T stage, and pathologic tumor-node-metastasis (TNM) stage according to the 7th edition of lung cancer staging system [13]. Survival and disease relapse were recorded on the basis of follow-up clinic or telephone. After surgery, 74 patients were followed up in the clinic using chest computed tomography, physical examination, routine blood tests, serum tumor marker tests, brain magnetic resonance imaging, bone scanning, and ultrasonography of the neck and abdomen. Eight patients were lost to follow-up. Follow-up evaluations were performed every 3 months during the first year, every 4 months during the second year, and then every 6 months. The median follow-up period was 22.5 months (range, 3–63 months) for the 74 patients with follow-up records.

### 2.4. Statistical Analysis

The follow-up period was defined as the time from surgery to the last observation for censored cases, or death for complete observations. Relapse-free survival (RFS) was defined as the time from the date of primary surgery to the date of relapse, or September 2014. Patients without a study end date and who were lost to follow-up were considered censored. The statistical analysis was performed using GraphPad Prism version 5.0 software (Graphpad Software, Inc., California, USA). The unpaired Student's *t*-test was used for between-group comparisons. Survival curves were plotted using the Kaplan-Meier method. Univariate and multivariate analyses were performed using the Cox proportional hazards model. A two-tailed *p* value < 0.05 was considered to be statistically significant.

## 3. Results

### 3.1. Patient Characteristics

A total of 82 early-stage non-small cell lung cancer patients and 81 late-stage non-small cell lung cancer patients were enrolled in this retrospective study. Thirty-one female and 51 male patients were enrolled in the early-stage group (*N* = 82). Eighteen female and 63 male patients were enrolled in late-stage group (*N* = 81). The general characteristics of the subjects that participated in the study are presented in Table 1.

### 3.2. Detection of Serum GRP78

The mean ± standard error (SE) GRP78 level was 326.5 ± 49.77 in the early-stage lung cancer patients. This level was significantly lower compared with the level in the late-stage lung cancer patients (*p* = 0.0001).

### 3.3. Correlation of GRP78 with Various Clinicopathological Factors

The results indicated that there were no significant associations between GRP78 expression and the clinicopathological variables age, gender, pathological T, and pathological stage for the early-stage patients (Table 2).

### 3.4. Prognostic Factors for Non-Small Cell Lung Cancer Patients

We performed a univariate analysis of the clinicopathological factors to examine the factors that predict prognosis (Table 3). The median value of GRP78 protein was 127.6 ng/mL for early-stage non-small cell lung cancer patients. Therefore, patients were divided into two groups (GRP78 value > 127.6 ng/mL and GRP78 value ≤ 127.6 ng/mL). Among the factors analyzed, age, gender, differentiation, pathological T, and GRP78 level were significant prognostic factors (*p* = 0.0052, *p* = 0.0152, *p* = 0.0113, *p* = 0.0337, and *p* = 0.0334, resp., Table 3).

Five factors were subjected to multivariate analysis to identify independent prognostic factors: age, gender, differentiation, pathological T, and GRP78 level. The multivariate analysis revealed that gender, differentiation, and GRP78 level were three independent prognostic factors in these non-small cell lung cancer patients (Table 4).

### 3.5. Survival Analysis

Data from 74 patients were included in the survival analysis. The median follow-up time was 22.5 months for the entire cohort. The median RFS was 19 months for the 74 patients. The results indicated that the 1-, 2-, 3-, and 5-year survival rates for the 74 lung cancer patients were 90.14%, 79.33%, 70.79%, and 64.35%, respectively.

**Figure 1:**
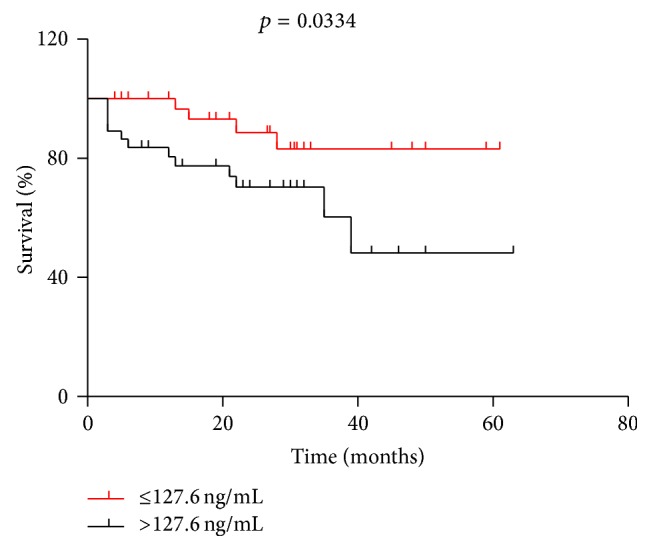
Kaplan-Meier survival curves for early-stage non-small cell lung cancer patients (*n* = 74), in relation to GRP78 protein expression. The survival of patients with higher GRP78 expression was significantly lower (*p* = 0.0334).

The results for the Kaplan-Meier survival curve analysis indicated that the patients with a higher GRP78 expression had shorter survival times compared with patients with a lower GRP78 expression (median overall survival, 39 versus 48.7 months) (RR: 0.3419, 95% confidence interval (CI): 0.1272–0.9190; *p* = 0.0334) (Figure 1). However, the difference in relapse-free survival (RFS) between the low GRP78 expression (≤127.6 ng/mL) group and the high GRP78 expression (>127.6 ng/mL) group was not significant (*p* = 0.1585, Figure 2).

## 4. Discussion

A robust induction of GRP78 occurs in many malignancies, including prostate cancer, breast cancer, hepatocellular carcinoma, and colon cancer [14–17]. Recent studies demonstrate that GRP78 expression is correlated with poor prognosis in melanoma [18]. Wang et al. found that a high level of GRP78 is more common in patients with high-grade lung cancer [19]. Uramoto et al. reported that a positive expression of GRP78 is a significant factor that indicates a favorable prognosis [6]. The findings of Zheng et al. indicated that GRP78 is an effective and objective marker for aggressive behavior and poor prognosis in gastric carcinoma patients [20]. Controversy persists regarding the prognosis indicated by various GRP78 levels in malignancies. Until now, no studies have detected GRP78 in the serum of non-small cell lung cancer patients, the etiology of lung cancer is complex, and newer prognostic markers are needed for management of this lethal disease. We thus analyzed GRP78 expression in the serum of lung cancer patients and have provided results that indicate its potential as a prognostic biomarker for this serious malignant disease.

Compared with early-stage non-small cell lung cancers, we found significantly elevated levels of serum GRP78 expression in late-stage non-small cell lung cancers. This result was consistent with the results of Wang et al.'s study [19]. The regulation and expression of GRP78 have been associated with shorter overall survival times [21–23]. Our results were consistent with those of previous reports. High GRP78 expression may be a predictor of a shorter overall survival time.

Novel ER stress markers for drug responsiveness prediction are emerging as there has been an increasing recognition of an association between ER stress and human cancer, and an improved understanding of the diverse underlying molecular mechanisms [24]. GRP78 has been extensively documented to confer resistance against a wide range of therapies including chemotoxic drugs, antihormonal agents, DNA damaging agents, antiangiogenesis drugs, and chromatin-modifying drugs, as well as radiation therapy [25]. Several studies have shown that GRP78 conferred resistance against Adriamycin-mediated apoptosis in cancer cells [26]. Jiang et al. suggested that knockdown of GRP78 can enhance the sensitivity of melanoma to chemotherapy drugs [27]; removing the tumor protection provided by ERS may enhance the sensitivity of chemotherapy drugs [28]. Roller and Maddalo found that GRP78 is often overexpressed in several types of cancers refractory to conventional therapy. GRP78 therefore may be not only a good biomarker to predict response to therapy, but also an appealing target for more selective chemotherapy approaches [29]. This may be one viable means of treating non-small cell lung cancer. However, a large group of patients with long-term follow-up is needed, and more cellular experiments should be performed, to confirm this conclusion. These approaches present exciting new opportunities for biological and clinical lung cancer research.

There were several limitations to our study that may have affected our results. First, this was a retrospective study. Second, the study sample size was small. Significant correlations with the clinicopathological parameters may have resulted if a greater number of patients had been included in the study. Third, we did not closely examine the response to chemotherapy and/or radiotherapy in the late-stage non-small cell lung cancer patients.

## 5. Conclusion

In conclusion, herein, our study was the first to detect the presence of GRP78 in serum of non-small cell lung cancer patients. The expression of GRP78 was highly enriched in the late-stage lung cancer patients and may be an important prognostic marker for non-small cell lung cancer. There were no significant correlations with clinicopathological parameters. Larger and more detailed follow-up studies will be required to confirm this finding and to determine if additional clinicopathological correlations with GRP78 expression can be identified. Future studies will be required to determine whether GRP78 levels can be used to guide the treatment of non-small cell lung cancer patients.

## Figures and Tables

**Figure 2:**
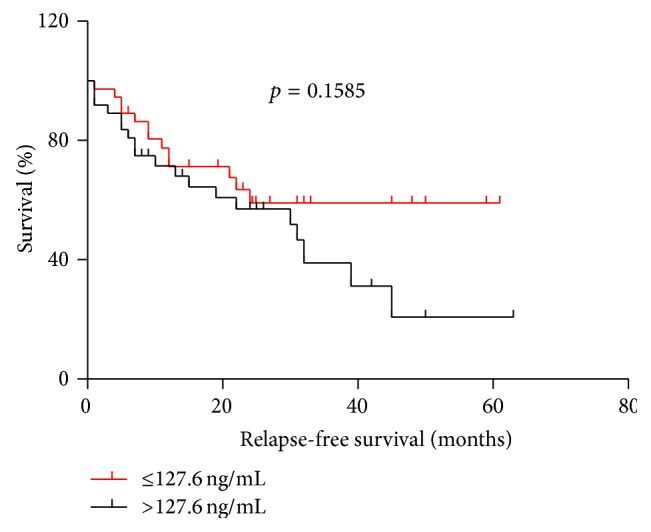
There were no significant differences in relapse-free survival (RFS) time between low GRP78 expression (≤127.6 ng/mL) and high GRP78 expression (>127.6 ng/mL) (*p* = 0.1585) levels.

**Table 1:** Characteristics of lung cancer patients.

Parameters	Early-stage group (*N* = 82)	Late-stage group (*N* = 81)
Age (years, mean ± SD)	58.9 ± 10.2	58.2 ± 10.0
(Range)	36–76	34–81
Gender		
Male	51	63
Female	31	18
Stage		
I	38	—
II	10	—
III	34	—
IV	—	81
Differentiation		**—**
Well and moderate	40	**—**
Poor	42	** —**
Treatment		
Surgery	82	** —**
Chemoradiotherapy/radiotherapy	—	81

SD: standard deviation.

**Table 2:** Correlation of GRP78 with various clinicopathological factors for early-stage lung cancer patients.

Parameters	Mean (pg/mL) ± SE	*p* value
Age (years)		
≤60	232.9 ± 54.22	0.0534
>60	424.8 ± 82.58	
Gender		
Male	313.3 ± 61.08	0.7350
Female	348.3 ± 86.23	
pT		
T1-T2	118.91 ± 426.24	0.2483
T3	300.52 ± 441.76	
Stage		
I-II	295.3 ± 59.96	0.4600
III	370.6 ± 85.65	
Differentiation		
Well and moderate	363.2 ± 81.65	0.4755
Poor	291.6 ± 58.77	

SE: standard error, pT: pathological tumor.

**Table 3:** Prognostic factors for patients with early-stage non-small cell lung cancers (*n* = 74). Univariate analysis.

Factor	Univariate analysis		*p* value
	95% CI	HR	
Age (>60 years versus ≤60 years)	0.085–0.649	0.235	0.0052
Gender (male versus female)	1.273–9.565	3.489	0.0152
Differentiation (well and moderate versus poor)	1.336–9.653	3.592	0.0113
pTNM stage after the surgery (stages I-II versus stage IIIa)	0.229–1.816	0.645	0.4064
pT (T1-2 versus T3)	0.0484–0.886	0.207	0.0337
pN (N0 versus N1-2)	0.265–1.924	0.714	0.5114
GRP78 level (≤127.6 ng/mL versus >127.6 ng/mL)	0.127–0.919	0.342	0.0334

HR: hazard ratio, pTNM: pathological tumor-node-metastasis, pT: pathological tumor, pN: pathological node.

**Table 4:** Multivariate analysis results.

Factor	95% CI	Risk ratio	*p* value
Age (>60 years versus ≤60 years)	0.822–10.104	2.88	0.098
Gender (male versus female)	0.038–0.855	0.181	0.031
Differentiation (well and moderate versus poor)	0.046–0.629	0.171	0.008
pT (T1-2 versus T3)	0.161–1.488	0.489	0.208
GRP78 level (≤127.6 ng/mL versus >127.6 ng/mL)	0.088–0.979	0.294	0.046

pT: pathological tumor.

## Abbreviations

GRP78: Glucose-regulated protein 78
BIP: Immunoglobulin heavy chain binding protein
ER: Endoplasmic reticulum
ELISA: Enzyme-linked immune sorbent assay
TNM: Tumor-node-metastasis
OS: Overall survival
RFS: Relapse-free survival.

## References

[1] Jemal A., Bray F., Center M. M., Ferlay J., Ward E., Forman D. (2011). Global cancer statistics. *CA Cancer Journal for Clinicians*.

[2] Sant M., Aareleid T., Berrino F. (2003). EUROCARE-3: survival of cancer patients diagnosed 1990–94—results and commentary. *Annals of Oncology*.

[3] Korpanty G., Smyth E., Carney D. N. (2011). Update on anti-angiogenic therapy in non-small cell lung cancer: are we making progress?. *Journal of Thoracic Disease*.

[4] McKeage M. J., Jameson M. B., AS1404-201 Study Group Investigators (2010). Comparative outcomes of squamous and non-squamous non-small cell lung cancer (NSCLC) patients in phase II studies of ASA404 (DMXAA)—retrospective analysis of pooled data. *Journal of Thoracic Disease*.

[5] Shash E., Peccatori F. A., Azim H. A. (2011). Optimizing the use of epidermal growth factor receptor inhibitors in advanced non-small-lung cancer (NSCLC). *Journal of Thoracic Disease*.

[6] Uramoto H., Sugio K., Oyama T. (2005). Expression of endoplasmic reticulum molecular chaperone Grp78 in human lung cancer and its clinical significance. *Lung Cancer*.

[7] Ni M., Zhang Y., Lee A. S. (2011). Beyond the endoplasmic reticulum: atypical GRP78 in cell viability, signalling and therapeutic targeting. *Biochemical Journal*.

[8] Kern J., Untergasser G., Zenzmaier C. (2009). GRP-78 secreted by tumor cells blocks the antiangiogenic activity of bortezomib. *Blood*.

[9] Bánhegyi G., Baumeister P., Benedetti A. (2007). Endoplasmic reticulum stress. *Annals of the New York Academy of Sciences*.

[10] Kimata Y., Kohno K. (2011). Endoplasmic reticulum stress-sensing mechanisms in yeast and mammalian cells. *Current Opinion in Cell Biology*.

[11] Fu Y., Lee A. S. (2006). Focused eeview: ER stress and cancer glucose regulated proteins in cancer progression, drug resistance and immunotherapy. *Cancer Biology & Therapy*.

[12] Quinones Q. J., de Ridder G. G., Pizzo S. V. (2008). GRP78, a chaperone with diverse roles beyond the endoplasmic reticulum. *Histology and Histopathology*.

[13] Edge S., Byrd D., Compton C. (2010). *American Joint Committee on Cancer, American Cancer Society: AJCC Cancer Staging Manual*.

[14] Daneshmand S., Quek M. L., Lin E. (2007). Glucose-regulated protein GRP78 is up-regulated in prostate cancer and correlates with recurrence and survival. *Human Pathology*.

[15] Fernandez P. M., Tabbara S. O., Jacobs L. K. (2000). Overexpression of the glucose-regulated stress gene GRP78 in malignant but not benign human breast lesions. *Breast Cancer Research and Treatment*.

[16] Shao Q., Ren P., Li Y. (2012). Autoantibodies against glucose-regulated protein 78 as serological diagnostic biomarkers in hepatocellular carcinoma. *International Journal of Oncology*.

[17] Takahashi H., Wang J.-P., Zheng H.-C., Masuda S., Takano Y. (2011). Overexpression of GRP78 and GRP94 is involved in colorectal carcinogenesis. *Histology and Histopathology*.

[18] Guan M., Chen X., Ma Y. (2015). MDA-9 and GRP78 as potential diagnostic biomarkers for early detection of melanoma metastasis. *Tumor Biology*.

[19] Wang Q., He Z., Zhang J. (2005). Overexpression of endoplasmic reticulum molecular chaperone GRP94 and GRP78 in human lung cancer tissues and its significance. *Cancer Detection and Prevention*.

[20] Zheng H.-C., Takahashi H., Li X.-H. (2008). Overexpression of GRP78 and GRP94 are markers for aggressive behavior and poor prognosis in gastric carcinomas. *Human Pathology*.

[21] Kuroda K., Horiguchi A., Asano T. (2011). Glucose-regulated protein 78 positivity as a predictor of poor survival in patients with renal cell carcinoma. *Urologia Internationalis*.

[22] Matsuo K., Gray M. J., Yang D. Y. (2013). The endoplasmic reticulum stress marker, glucose-regulated protein-78 (GRP78) in visceral adipocytes predicts endometrial cancer progression and patient survival. *Gynecologic Oncology*.

[23] Papalas J. A., Vollmer R. T., Gonzalez-Gronow M. (2010). Patterns of GRP78 and MTJ1 expression in primary cutaneous malignant melanoma. *Modern Pathology*.

[24] Zheng Y.-Z., Cao Z.-G., Hu X., Shao Z.-M. (2014). The endoplasmic reticulum stress markers GRP78 and CHOP predict disease-free survival and responsiveness to chemotherapy in breast cancer. *Breast Cancer Research and Treatment*.

[25] Lee A. S. (2014). Glucose-regulated proteins in cancer: molecular mechanisms and therapeutic potential. *Nature Reviews Cancer*.

[26] Tsai H.-Y., Yang Y.-F., Wu A. T. (2013). Endoplasmic reticulum ribosome-binding protein 1 (RRBP1) overexpression is frequently found in lung cancer patients and alleviates intracellular stress-induced apoptosis through the enhancement of GRP78. *Oncogene*.

[27] Jiang C. C., Mao Z. G., Avery-Kiejda K. A., Wade M., Hersey P., Zhang X. D. (2009). Glucose-regulated protein 78 antagonizes cisplatin and adriamycin in human melanoma cells. *Carcinogenesis*.

[28] Xia F., Xu J. C., Zhang P. (2014). Glucose-regulated protein 78 and heparanase expression in oral squamous cell carcinoma: correlations and prognostic significance. *World Journal of Surgical Oncology*.

[29] Roller C., Maddalo D. (2013). The molecular chaperone GRP78/BiP in the development of chemoresistance: mechanism and possible treatment. *Frontiers in Pharmacology*.

